# Memorials to King Edward

**Published:** 1911-12-16

**Authors:** James Gildea

**Affiliations:** 11 Hogarth Road, London, S.W.


*To the Editor of* The Hospital.

### MEMORIALS TO KING EDWARD.

*To the Editor of* The Hospital.

Sir,—I am much obliged for the notice in your paper drawing the attention of the hospital Secretaries to the above, which, if followed, should materially help.

May I further venture to ask if you would favour me with a copy of your issues which give a list of those Hospital Memorials which have already been started?—

Yours truly, James Gildea.

11 Hogarth Road, London, S.W.

December 9.

[Lists of Hospital Memorials appeared in our issues of March 9 (pp. 669-670) and April 15 (p. 63).—Ed. The Hospital.]

